# Reduction of organic contaminants from industrial effluent using the advanced oxidation process, chemical coagulation, and green nanotechnology

**DOI:** 10.1038/s41598-024-65162-6

**Published:** 2024-07-02

**Authors:** Amany M. Naguib, Soha A. Abdel-Gawad, Ahmed S. Mahmoud

**Affiliations:** 1 Sanitary and Environmental Institute (SEI), Housing and Building National Research Center (HBRC), Giza, Egypt; 2 Chemistry Departments, Faculty of Science, Cairo University, Giza, Egypt; 3 Institute of Environmental Studies (IES), Arish University, North Sinai, Egypt

**Keywords:** Environmental toxicology, Fenton oxidation, Advanced oxidation process, Organic matter removal, COD removal, Sustainable development goals, Environmental sciences, Chemistry, Nanoscience and technology

## Abstract

Municipal wastewater treatment systems use the chemical oxygen demand test (COD) to identify organic contaminants in industrial effluents that impede treatment due to their high concentration. This study reduced the COD levels in tannery wastewater using a multistage treatment process that included Fenton oxidation, chemical coagulation, and nanotechnology based on a synthetic soluble COD standard solution. At an acidic pH of 5, Fenton oxidation reduces the COD concentration by approximately 79%. It achieves this by combining 10 mL/L of H_2_O_2_ and 0.1 g/L of FeCl_2_. Furthermore, the author selected the FeCl_3_ coagulant for the coagulation process based on the best results of comparisons between different coagulants. At pH 8.5, the coagulation dose of 0.15 g/L achieved the maximum COD removal efficiency of approximately 56.7%. Finally, nano bimetallic Fe/Cu was used to complete the degradation and adsorption of the remaining organic pollutants. The XRD, SEM, and EDX analyses proved the formation of Fe/Cu nanoparticles. A dose of 0.09 g/L Fe/Cu NPs, 30 min of contact time, and a stirring rate of 200 rpm achieve a maximum removal efficiency of about 93% of COD at pH 7.5. The kinetics studies were analyzed using pseudo-first-order P.F.O., pseudo-second-order P.S.O., and intraparticle diffusion models. The P.S.O. showed the best fit among the kinetic models, with an R^2^ of 0.998. Finally, the authors recommended that technique for highly contaminated industrial effluents treatment for agriculture or industrial purposes.

## Introduction

A variety of sources, including households, businesses, industries, and agriculture, produce wastewater. Wastewater encompasses any water that has undergone use and discharge, including that from toilets, sinks, showers, washing machines, and dishwashers. Stormwater runoff, which is water that runs off streets, parking lots, and other surfaces during rain events, can also produce wastewater. Industrial processes and agricultural activities can also generate wastewater that may contain pollutants and contaminants. Proper treatment and management of wastewater is important to protect public health and the environment. As the population grows and water consumption increases, so does the amount of wastewater produced. Wastewater can contain a variety of organic and inorganic substances, including nutrients, pathogens, and pollutants. The presence of organic molecules in wastewater, which may raise the level of COD, is especially common in waste materials like industrial waste and human waste. High COD levels can indicate that the wastewater contains a high number of organic pollutants, which can be harmful to the environment if not properly treated. Organic acids, alcohols, mono and poly-aromatic hydrocarbons, phenols, aldehydes, ketones, and other halogenated organic materials can be made from industrial effluents like wastewater from pharmaceutical manufacturing, solvent use, oil refining, and even household wastewater^1^. Wastewater treatment can employ a variety of techniques, including chemical, biological, and physical methods^2^. Physical separation of large, suspended particles, temperature control, and oil separation are just a few examples of the various physical treatment approaches. Additionally, there are several types of biological treatments that are both aerobic and anaerobic. Chemical treatments consider adsorption techniques, sophisticated oxidation procedures, coagulation and flocculation methods, and nanotechnology^3^. Developing countries are trying to explore low-cost wastewater treatment technologies. One of these technologies is biological treatment with anaerobic and catalytic sludge, which is a more cost-effective option than other methods. It is a natural process that uses microorganisms to break down organic materials in wastewater. However, completing the treatment process may take some time. Therefore, developing countries are trying to find effective and faster solutions. Therefore, developing countries are trying to reach effective and faster solutions. Advanced oxidation methods, such as the Fenton process, can be very effective in reducing organic compounds in wastewater. The Fenton process adds hydrogen peroxide and ferrous ions to wastewater, producing hydroxyl radicals that can break down organic pollutants. This process is particularly useful for treating wastewater containing persistent organic pollutants that are difficult to remove using conventional treatment methods. However, it is important to note that the Fenton process is a sustainable solution that can help protect the environment and public health^4^. It has high oxidation capabilities and oxidizes almost all organic contaminants. Municipal wastewater and industrial wastewater undergo treatment using the Fenton oxidation method. (HO^⋅^) is produced when Fe^2+^ ions interact with H_2_O_2_. All experiments apply acidic pH conditions to prevent iron precipitation. Nowadays, one of the techniques used to treat wastewater and other types of environmental pollutants is nanotechnology. Nowadays people widely use nanoparticles as anti-microbes because they effectively limit bacterial development and act as antiseptics^5,6^. Furthermore, the green synthesized bimetallic Fe/Cu particles have proven effective in the treatment of wastewater nanoparticles due to being more stable and capable of rapidly removing organic and inorganic materials from water solutions at a low cost. Furthermore, the green synthesized bimetallic Fe/Cu particles have proven effective in the treatment of wastewater nanoparticles due to being more stable and capable of rapidly removing organic and inorganic materials from water solutions at a low cost. The bimetallic particles have shown improved performance compared to monometallic particles in terms of catalytic activity and stability. Overall, the green synthesized bimetallic Fe/Cu particles offer a promising solution for wastewater treatment that is both environmentally sustainable and cost-effective^7,8^. The main goal of this study is to use the Fenton oxidation method, followed by coagulation-precipitation processes, and green nanoparticles in wastewater treatment.

## Experimental

### Chemicals

The chemicals used in this study include Ferric chloride hexahydrate GPR (FeCl_3_.6H_2_O, 98% pure, Koch- Light Laboratory Ltd Company), Ferrous chloride extra pure (Hydrate) (FeCl_2_ .XH_2_O, 98%, Loba chemie), Hydrogen Peroxide (H_2_O_2_, 50%, 30% w/w Piochem), Cupric sulphate anhydrous (CuSO_4_, 98%, LOBA CHEMIE), anhydrous primary- standard- grade Potassium Hydrogen Phthalate (C_8_H_5_KO_4_, 99.5% spectrum), Sodium borohydride (NaBH_4_, 99%, Loba chemie), Soft Kenian Black Tea (Al-Arosa), Ethanol (C_2_H_5_OH 95%, World co. for sub & med industries), Aluminum sulphate (Al_2_(SO_4_)_3_, 95% pure, Al-Ahram), Zinc sulfate (ZnSO_4_, 99.9% Win lab), potassium dichromate (K_2_Cr_2_O_7_ 99.5%, PROLABO), Solution, other concentrations were obtained by dilution of 0.1 M from H_2_SO_4_ (98%, Honeywell), and NaOH (99%, Oxford co.) was used for changing the pH along with deionized distilled water from Millipore (Elix) Instrument.

### The preparation of green bimetallic (Fe/Cu) nanoparticles

According to research by Zin et al. (2013) and Mahmoud et al. (2020 and 2021), the bimetallic Fe/Cu nanoparticles were developed^1,9–11^ as follows:

- Step 1. Preparation of reducing material: About 40 g of black tea have been added to 3 L of distilled water and heated for two hours at 200 °C. After cooling, the extracted solution was filtrated using filter paper number 1. Furthermore, about 50 mL of ethanol were mixed with the unfiltered tea to complete the dissolution of the phenolic mixtures and filtrated again. Finally, 10 g of NaBH_4_ was dissolved in the extract to increase reduction efficiency.
- Step 2. Preparation of the Iron Source: About 4 g of FeCl_3_.6H_2_O were added to one liter of a 5% ethanol–water mixture (0.12 M).
- Step 3. Preparation of the Cupper source: About 10 g of CuSO_4_ were added to one liter of the same iron source solution in a 5% ethanol–water mixture (0.06 M).
- Step 4. Reduction process: the extract solution was added to the mixture solution of [Fe (III)-Cu (II)] at a stirring rate of 150 rpm to form bimetallic Fe/Cu nanoparticles.
- Step 5. Nano-formation: The colour of the solution was altered from faint green to black. The black precipitate formed and precipitated when the mixture solution [Fe (III)-Cu (II)] was completely reduced by extracted phenolic compounds. The black precipitate becomes suitable for use after washing with 50 mL of ethanol, centrifugation, and drying for two hours at a fixed temperature of 150 °C^12^.

### Prepare a standard solution of organic matter represented in COD

A 1000-mL volumetric container was filled to the required quantity after 0.425 g of potassium hydrogen phthalate had been dissolved to create a COD standard solution. This is in conformity with Standard Methods for the Examination of Water and Wastewater, 24th edition. The prepared COD standard solution has a concentration of 500 mg _(O2)_/L COD.

### Batch studies

Batch studies included 3 steps (A-B-C) are shown in Table 1.

- Step A: In the Fenton process, the degradation of organic pollutants represented by COD was investigated by the Fenton process in batch studies. Reduction of organic compounds was investigated at different pH values (1, 2, 3, 4, 5, and 6), a various moles of ferrous ion (0.0003, 0.0007, 0.0011, 0.0015, 0.0019, and 0.0023 mol) , a different milliliter of hydrogen peroxide (2, 4, 6, 8, 10, and 12 mL) , and different concentration (200, 400, 600, 800, and 1000 ppm) at a contact time of 3 min of mixing at 150 rpm, followed by 30 min of settling, then filtration^5,13^.
- Step B: In the coagulation process, the treatment was studied with three common coagulants, namely Al_2_(SO_4_)_3_, FeCl_3_⋅6H_2_O, and ZnSO_4_. Reduction of organic matter was studied at pHs (6.5, 7, 7.5, 8, 8.5, and 9) and a known dose of coagulants (0.05, 0.1, 0.15, 0.2, and 0.25) at a contact time of 3 min of mixing at 150 rpm, 20 min of floc formation, followed by an hour of settling and filtration.
- Step C: The treatment was done with bimetallic Fe/Cu nanoparticles. The removal of organic compounds was tested at various pHs (6, 6.5, 7, 7.5, 8, 8.5, and 9), doses of adsorbent (0.01, 0.02, 0.03, 0.04, 0.05, 0.06, 0.07, 0.08, and 0.09 g/L), and stirring rates (100, 150, 200, 250, 300, 350, and 400 rpm) at various times (10, 20, 30, 40, 50, and 60 min). After complete precipitation, the filtration process occurred using zeolite, and the final (COD) concentrations were measured^14^.
  1 $${\text{R}}\left( \% \right) \, = \frac{{{\text{Co}} - {\text{ Ce}}}}{{{\text{Co}}}} \times 100$$
  2 $${\text{Q}}_{{\text{e}}} \left( {{\text{mg}}/{\text{mg}}} \right) \, = \frac{{\left( {{\text{Co}} - {\text{ Ce}} } \right){\text{V}}}}{{\text{m}}}$$
  Where R (%) is the removal percentage determined by Eq. (1) and the amount of organic matter represented by COD reduced by the weight of nanoparticles determined by Eq. (2), C_o_ is the concentration at the beginning (ppm), C_e_ is the concentration at equilibrium in solution (ppm), Q_e_ is the adsorption capacity at equilibrium (mg/mg), V is the volume of aqueous solution (L), and m is the mass of the adsorbent after drying (mg)^14^.

**Table 1:** Review for wastewater treatment using the Fenton oxidation process.

No	Type of wastewater	COD before (ppm)	COD elimination%	Optimum conditions	References
1	Batik cual wastewater	104	76.3%	Dosage of FeSO_4_.7H_2_O = 0.5 g/L, Dosage of H_2_O_2_ = 1 mL/L, Time = 24 h	15
2	Cosmetic wastewater	6968	95%	at pH 3, Dosage of H_2_O_2_ = 1 mL/L, and Dosage of Ferrous ion and Ferric ion = 0.75 g/L and time 40 min	16
3	Dyeing bath wastewater	–	(75.81, 78.03, and 78.14%) for the yellow, red, and blue dyes, consecutively	Dosage of H_2_O_2_ = 9 mL/L, FeSO_4_.7H_2_O_2_ = 0.12 g/L at pH 3 and time = 100 min	17
4	Launderette wastewater	704	63%	Dosage of Fe^3+^ = 0.04 g/L, Dosage of H_2_O_2_ = 1.3 mL/L at pH 3 after 15 min	18
5	Tannery wastewater	1200	70%	(Cat/OX = 35% at pH 3, Quantity of H_2_O_2_was a fixed was equal to 0.75*COD_0_ = 0.9 g/L (Fe^2+^ = 0.18, 0.315 g/L)	19
6	6% saline tannery wastewater	960	82%	Varying dosage of FeSO_4_.7H_2_O_2_ = (0.1, 0.3 g/L). dosage of H_2_O_2_ = (0.4, 0.8 g/L), at pH 4	20
7	Tannery wastewater	4000	71%	H_2_O_2_: FeSO_4_7H_2_O was (3.75: 0.1) g/L. Time = 4 h at pH 3.5	21
8	Synthetic wastewater	480—1200	70%	Dosage of H_2_O_2_ = 1 g/L and Dosage of FeSO_4_.7H_2_O_2_ = 0.5 g/L for concentration of phenol = 200 ppm and Dosage of H_2_O_2_ = 3 g/L and Dosage of FeSO_4_.7H_2_O_2_ = 1.5 g/L for concentration of phenol = 500 mg/L.Ratio of [Fe^2+^]/ [H_2_O_2_] (mol/mol) = 0.11. pH range (2.3 to 2.8)	22
9	Pesticide-containing wastewater	1540	–	The optimum cases for (MWEUV) lamps /Fenton method was at pH 5, Dosage of Fe^2+^ = 0.2 g/L, Dosage of H_2_O_2_ = 14.2 g/L and Time = 2 h	23
10	Textile wastewater	202	68%		24
11	Landfill leachate	5000	65%	Dosage of Fe^2+^ = 10 g/L and Dosage of H_2_O_2_ = 48 g/L	25

### Characterization of green bimetallic (Fe/Cu) nanoparticles

X-ray diffraction (XRD) was used to study a bimetallic Fe/Cu nanoparticle. A Philips XRG 3100 diffractometer, manufactured by the Netherlands-based Philips Electronics Company, was used for the XRD investigation. The diffractometer uses a graphite monochromator and copper K-alpha radiation to produce x-rays with a peak wavelength of 1.5418 Aº. The X-ray voltage and current were adjusted to 40 kV and 40 mA, respectively, and the powder sample was put within a stainless-steel holder. At a rate of 0.0167/sec, the XRD diffraction angle (2θ) changed from 0 to 70. In this instance, XRD was used to examine the bimetallic Fe/Cu nanoparticle. XRD is a widely utilized method to evaluate the crystal structure of materials. The Microvision (particle size measuring) model VGA-410 France was utilized by the CID Company for Pharmaceutical Industries in Egypt to conduct an analysis of the particle size distribution for dried samples. The collected data showed that 94% of the Fe/Cu powder was 50 nm. Samples were moved from 0 to 30 m at a rate of 0.02 m. Furthermore, the use of a scanning electron microscope (SEM) to examine the bimetallic Fe/Cu nanoparticle Specifically, the Philips Quanta 250 FEG SEM, which is manufactured by Philips Electronics in the United States, was used for this purpose. The SEM operates at a magnification of 160,000x, allowing for high-resolution imaging of the nanoparticle. SEM is a powerful tool for examining the surface morphology and composition of materials, and in this case, it was used to gain insights into the structure and properties of the bimetallic Fe/Cu nanoparticle. The SEM examination was performed at the National Research Center in Egypt. Finally, EDAX examination is a method used to determine the composition of the bimetallic Fe/Cu nanoparticles. Energy Dispersive X-ray Analysis, or EDAX for short, is a method that is used to ascertain a sample's elemental composition in combination with scanning electron microscopy, or SEM. The elemental makeup of the sample may be ascertained by detecting and analyzing the X-rays produced when an electron beam is directed towards it. In the case of the bimetallic Fe/Cu nanoparticles, the EDAX examination would provide information on the relative amounts of iron and copper present in the sample. This information is important for understanding the properties and behavior of nanoparticles in various applications^26^. Using the model VGA-410 France and the µ-tech manufacturing facility at CID Business for Pharmaceutical Industries, Egypt, a particle size distribution test was performed on green bimetallic Fe/Cu NPs dried powder^3^.

### Point of zero charge

To produce 0.1 m of KCl solution, about 7.455 g of KCL was dissolved in 1 L of distilled water. A 100 mL Erlenmeyer flask was filled with precisely 20 mL of the produced solution, and 1 N H2SO4 or 1 N NaOH (pHi) was used to change the pH values from 2 to 12. Separately, 0.1 g of Fe/Cu NPs were added to the modified flasks, and they were allowed to sit at lab temperature for 24 h. Using an AD 8000-Adwa pH metre, the final pH was determined (pHf). Three experiments yielded average pH changes after nano alterations, and all standard deviation values were within ± 0.02. Plotting the relationship between ΔpH values (final pH − initial pHi) and initial pH values (pHi) yields the point of zero charges of Fe/Cu NPs.)^27^.

### Kinetic studies

At a temperature of 25 °C, bimetallic nanoparticles (Fe/Cu) were added to synthetic wastewater at varying times to determine the optimum time to achieve equilibrium. The amount of organic matter represented by COD that was reduced at a given time (t) was determined by the Eq. (3).

3 $${\text{Q}}_{{\text{t}}} = \frac{{\left( {{\text{Co}} - {\text{Ct}}} \right){\text{V}}}}{{\text{W}}}$$

where Q_t_ (mg/mg) is the amount of organic matter represented by (COD) reduced at a given time as determined by Eq. (2), C_o_ is the concentration at the beginning of the reaction (mg/L), C_t_ is the concentration (mg/L) at the given time (t), V is the volume of the solution (L), and W is the mass of the sorbent^28^.

### Quality assurance

In this investigation, all experiments were carried out in triplicate. During the testing, blank samples simply containing COD standard solution and nanopowder were used. A month before the measurements, the apparatus and instruments underwent calibration, and the uncertainty factors were estimated for COD test. IBM SPSS Statistics 25 and Microsoft Office 365 were used for all statistical analyses. Also, the Quillbot program checked the grammar and English. The Turnitin plagiarism checker was used.

## Results and discussion

Wastewater contains a variety of organic compounds. These organic molecules can be characterized using biochemical oxygen demand (BOD) and COD methods. COD is described as the quantity of oxygen equivalent consumed by a powerful oxidizer during the chemical oxidation of organic materials. Batch studies used the Fenton process followed by the coagulation/precipitation process followed by iron/copper bimetallic nanoparticles under various operational parameters such as pH, dose (g/L) of FeCl_2_, H_2_O_2_, adsorbent material, contact time, and initial concentration.

### Characterization of green bimetallic nanoparticles (Fe/Cu)

#### XRD and SEM characterization

**Figure 1:**
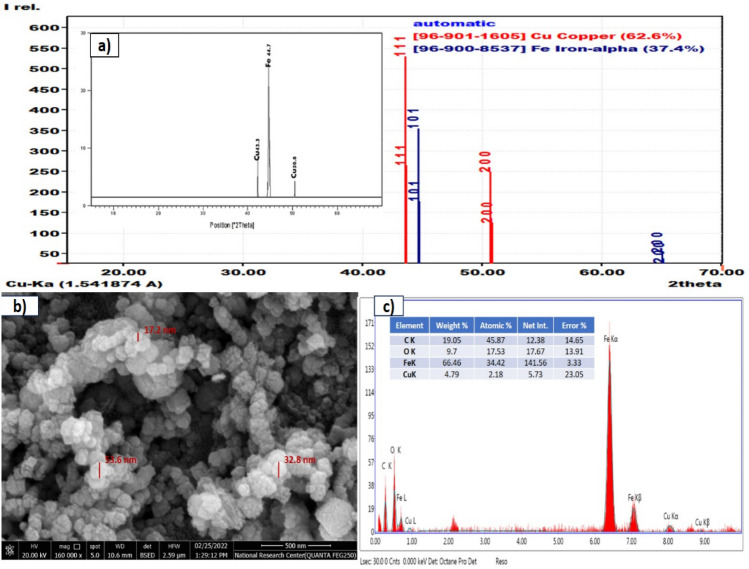
(**A**) X-ray diffraction for bimetallic Fe/Cu nanoparticles, (**B**) SEM analysis for bimetallic Fe/Cu nanoparticles, and (**C**) EDAX analysis for bimetallic Fe/Cu nanoparticles.

The XRD pattern Fig. 1a with (2θ) measurements ranging between 5 and 70 showed the formation of synthesized bimetallic nanoparticles (Fe/Cu) with referenced alfa iron [96-900-8537] and Cu [96-901-1605]. Alfa iron has two main identified peaks at 2θ equal 44.65 (101), and 64.98° (200). The main identified peaks of Cu were 43.3° (111) and 50.8° (200)^29^.

An SEM picture of semi-spherical bimetallic Fe/Cu NPs nanoparticles is shown in Fig. 1b. The findings demonstrate that the particles have an uneven surface structure, with sizes ranging from 10 to 50 nm. The produced nanoparticles were evident in the SEM picture. The presence of many pores enhances the mobility and accessibility of the contaminant's mass to the inner surface of the Fe/Cu synthesized bimetallic nanoparticles. Additionally, the SEM picture demonstrated how certain nanoparticles aggregated to create larger nanoclusters. This most likely happens because, as mentioned in the section on the preparation of bimetallic Fe/Cu nanoparticles, the Fe/Cu nanoparticles are made by combining FeCl_3_.6H_2_O solution with copper sulphate solution. Static magnetism and surface tension are primarily responsible for the interconnectivity of iron nanoparticles.

Figure 1c indicates the EDX method of the synthesized bimetallic nanoparticles (Fe/Cu), which shows the percentage of its components. Its results indicated the presence of elements C, O, Fe, and Cu with percentages for each weight of them of 19, 10, 66 and 5%, respectively. And the appearance of element C is due to the vehicles containing it that are formed during washing nanoparticles with ethanol. O signs can be due to the oxide composition in the outer layer of nanoparticles. This is due to the interaction of nanoparticles with water or air^30^.

#### Particle size distribution

**Figure 2:**
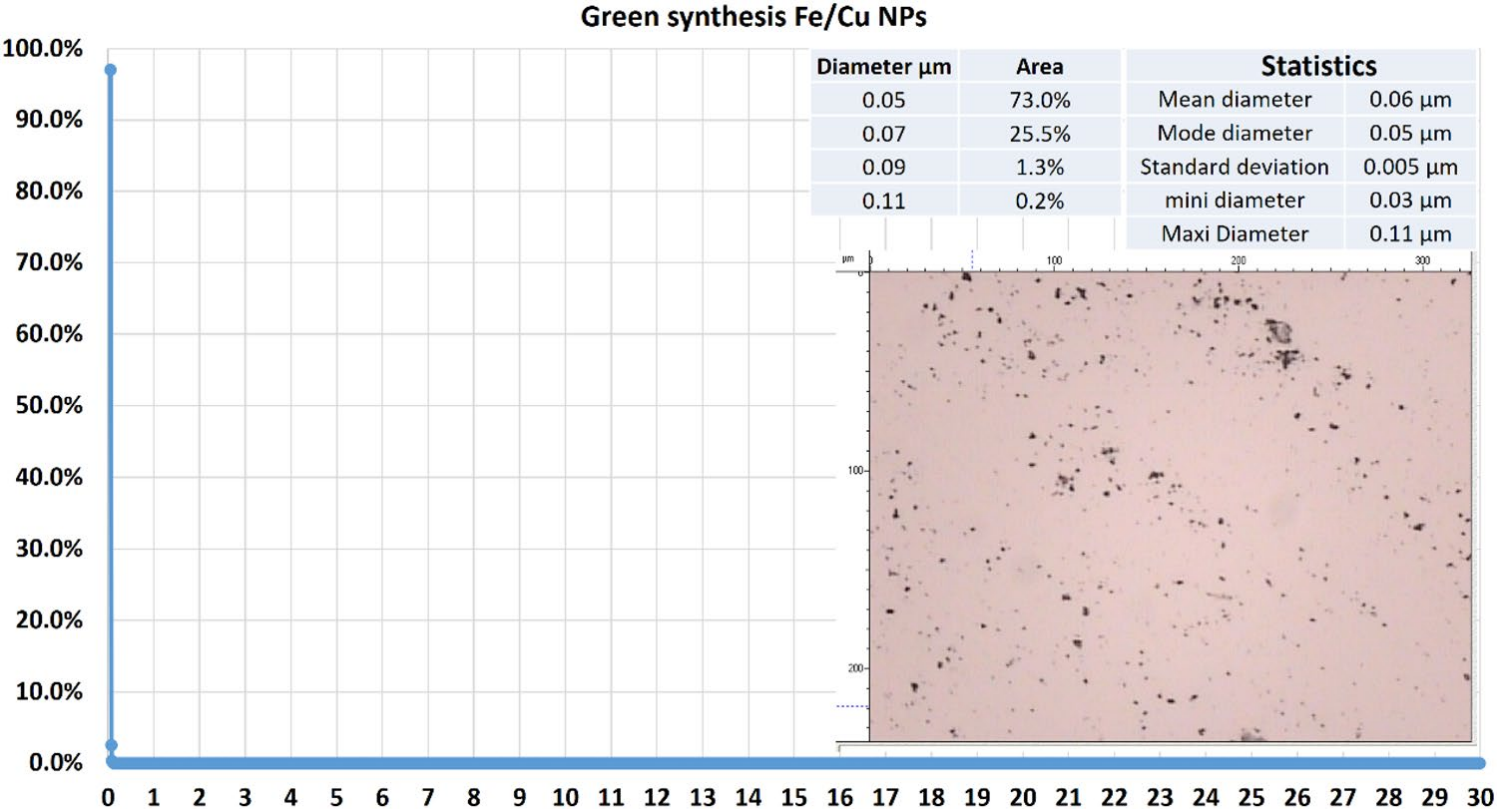
Particle size distribution for bimetallic Fe/Cu nanoparticles.

For Green Fe/Cu NPs, the particle size distribution was measured at a rate of 0.02 µm from 0 to 30 µm, as Fig. 2 illustrates. According to the findings, 73.0% of the sample was 50 nm and 99.8% of the sample was nanosized.

#### Point of zero charge

**Figure 3:**
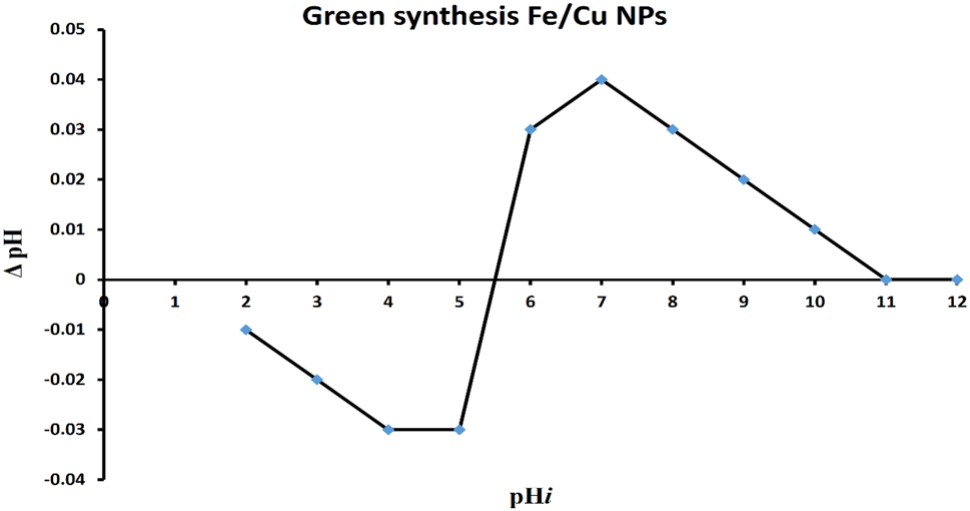
Point of Zero Charge for bimetallic Fe/Cu nanoparticles.

As shown in Fig. 3, the data gathered point of zero charges (pzc) values for Fe/Cu NPs were around 5.5, indicating consistency with the earlier findings. According to research done in 2019 by Hossam et al., the PZC for green Fe/Cu NPs prepared from Ficus Benjamina was around 4.9. Because there were significant amounts of carbons on the nano surface and the leftover extract solution had an impact, green nanomaterials had somewhat acidic values^3,27,31^

### Impact of operating conditions

#### Reduction of organic materials represented in COD by using the Fenton oxidation process (step a)

Fenton oxidation process can be used to destroy organic compounds. It is formed by a mixture of Ferrous chloride (Fe (II)) and hydrogen peroxide (H_2_O_2_). When hydrogen peroxide reacts with Ferrous chloride, reactive species called hydroxyl radicals are produced. A sequence of equations from (4) to (6) explains the classical radical reaction for H_2_O_2_ decomposition. Because electrical power from the surroundings rather than photochemical reactions energy drives the following sequence, it will be known as the thermal Fenton oxidation process. (The use of the word "thermal" does not indicate a high temperature.) In this order, every species that exists in solution under each oxidation state is assumed to be represented by Fe (II) and Fe (III)^32^.

4 $${\text{Fe }}\left( {{\text{II}}} \right) \, + {\text{ H}}_{{2}} {\text{O}}_{{2}} \to {\text{ Fe }}\left( {{\text{III}}} \right) \, + {\text{ OH}}^{ - } + {\text{ HO}}^{ \cdot }$$

5 $${\text{Fe}}\left( {{\text{III}}} \right) + {\text{H}}_{{2}} {\text{O}}_{{2}} \to {\text{Fe}}\left( {{\text{II}}} \right) + {\text{HO}}_{2}^{ \cdot } + {\text{ H}}^{ + }$$

6 $${\text{2H}}_{{2}} {\text{O}}_{{2}} \to {\text{ HO}}_{2}^{ \cdot } + {\text{ HO}}^{ \cdot } + {\text{ 2H}}_{{2}} {\text{O}}$$

The reaction takes place in an acidic environment rather than an alkaline environment because the reaction rate decreases due to the formation of amorphous ferric oxyhydroxide in an alkaline environment, as follows in Eqs. 4–6. Equation (4) showed that a labile ligand was occupying Fe (II), indicating an inner sphere electron transfer mechanism, and forming a hydroxide ion and a hydroxyl free radical as by-products. In Eq. (5), another hydrogen peroxide molecule reduces the ferric ion to the ferrous ion. As by-products, a hydroperoxyl free radical and a proton were formed. Then, the ferrous ion catalyst is remade. In Eq. (6), disproportionation of the hydrogen peroxide molecules occurs. And two oxygen radicals were formed. Hydroxide ions and protons are also produced as by-products and form water^32^.

##### The impact of pH on COD reduction

**Figure 4:**
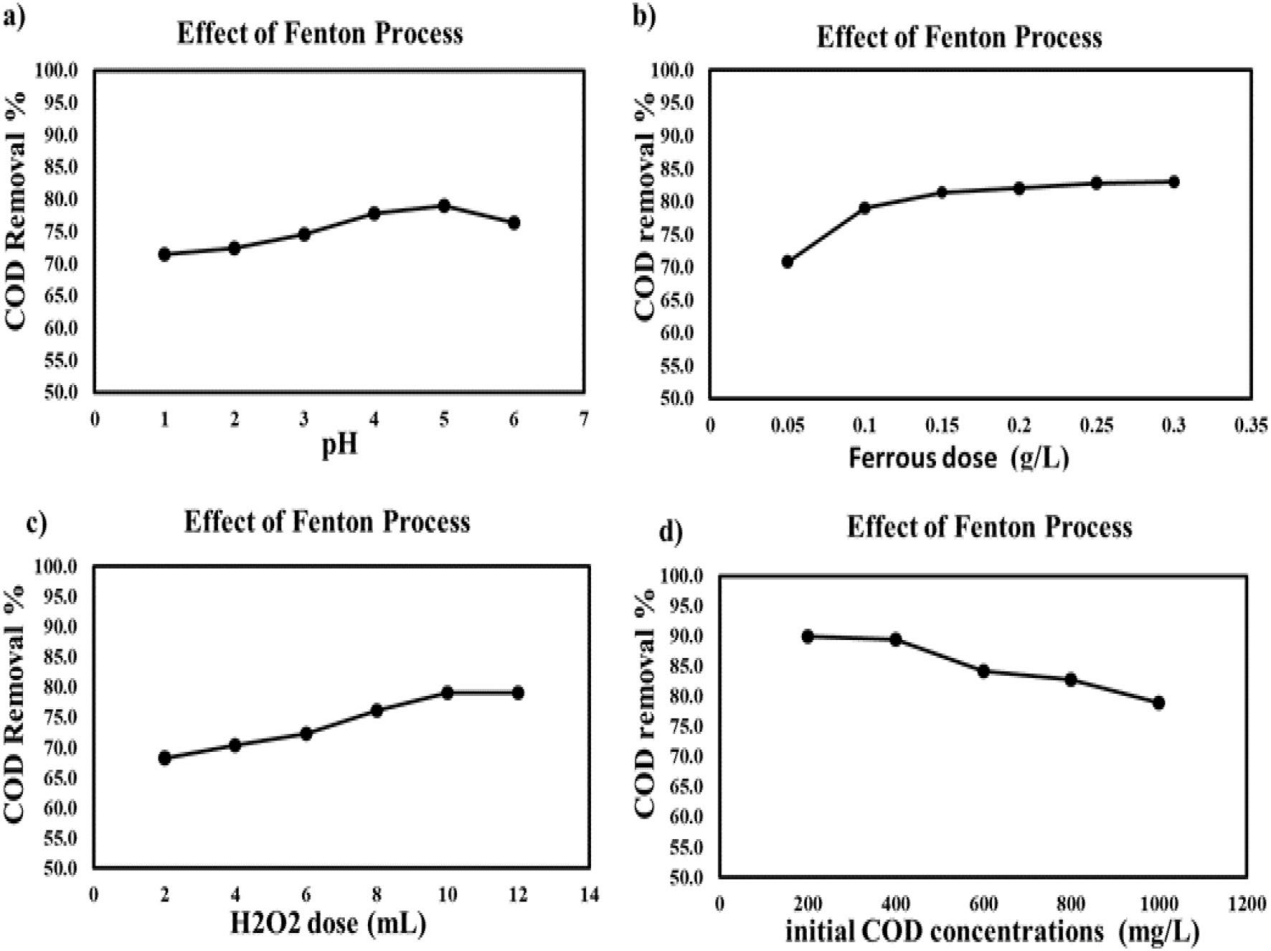
Impact of operating parameter on COD removal using Fenton process (**a**) impact of pH, (**b**) impact of ferrous dose, (**c**) impact of H_2_O_2_ dose, and (**e**) impact of initial concentration.

The reduction of organic materials represented by COD from synthetic wastewater was studied at various pHs (1, 2, 3, 4, 5, and 6). NaOH and sulfuric acid solutions were used to change the pH 0.1 g of FeSO_4_.7H_2_O and 10 mL of a 30% H_2_O_2_ solution were added successively to 1000 mL of synthetic wastewater. All the previous conditions at the time of contact 3 min of mixing at 150 rpm, followed by 30 min of settling, then filtering, and the COD contents of the beakers' supernatants were determined. At pH 5, the COD elimination percentage is effective. It was high (79.4%), as illustrated in Fig. 4a. In an acidic medium, when pH increases, COD removal percentages increase. However, under alkaline conditions, the reaction rate decreases due to the formation of ferric hydroxide^32^. At pH less than 5, the reduction of the ferric ion to the ferrous ion is slow. At a pH value greater than 5, H_2_O_2_ decomposes more slowly, fewer hydroxyl radicals are released, ferric oxyhydroxide (Fe-OOH) is formed, which reduces the rate of degradation, and iron precipitates as ferric hydroxide^33,34^.

##### The impact of ferrous dose

The impact of ferrous dose on the reduction of organic materials represented by COD from synthetic wastewater was studied between 0.05 and 0.3 g/L. 0.1 M NaOH and 0.1 M sulfuric acid solutions were used to change the pH, and 10 mL of a 30% H_2_O_2_ solution was added. Then, varying quantities of FeSO_4_·7H_2_O were put into the various beakers. The Jar test was used. All the previous conditions at the time of contact: 3 min of mixing at 150 rpm, followed by 30 min of settling, then filtering, and the COD contents of the beakers' supernatants were determined as illustrated in Fig. 4b. The optimal dosage was 0.1 g/L of FeSO_4_.7H_2_O. At high dosages of Fe^2+^, the oxidation of organic contaminants was difficult due to a phenomenon called the "scavenging effect". When the concentration of Fe^2+^ is too high, it can react with hydroxyl radicals (⋅OH) generated by the reaction between hydrogen peroxide (H_2_O_2_) and Fe^2+^, forming Fe^3+^ and hydroperoxyl radicals (⋅OOH). Hydroperoxyl radicals are less reactive than hydroxyl radicals and are not as effective in oxidizing organic contaminants. As a result, the scavenging effect reduces the overall concentration of hydroxyl radicals available for the oxidation of organic contaminants, making the process less efficient^35^.

##### The impact of H_2_O_2_ dose

Using various of (2, 4, 6, 8, 10, and 12) for studying the impact of H_2_O_2_ dose on the reduction of organic materials represented in COD from synthetic wastewater. The pH was altered using solutions of 0.1 M NaOH and 0.1 M sulfuric acid. At acidic pH 5, using FeCl_2_ doses of 0.1 g/L synthesized wastewater. Then, different doses of H_2_O_2_ were added to the beakers. The jar test was used. All the previous conditions at the time of contact: 3 min of mixing at 150 rpm, followed by 30 min of settling, then filtering, and the COD contents of the beakers' supernatants were determined. Figure 4c shows how the COD elimination rate increased from 68.2% to 97% as the H_2_O_2_ dosage was raised. When hydrogen peroxide was used as the oxidizing agent, hydroxyl radicals (HO⋅) formed. It changes ferrous (Fe^2+^) ions into Ferric (Fe^3+^) ions. Through the Fenton reaction, very reactive hydroxyl radicals are created. After that, it causes the oxidant to dissociate, attacking and getting rid of the organic pollutants. The process of creating radicals becomes less effective. The decrease in organic pollutants does not increase at large H_2_O_2_ dosages (over 10 mL/L). It forms (HO_2_⋅) when it combines with excess hydrogen peroxide hydroxyl radicals. It creates less reactive radicals than HO⋅ and is a less active oxidizing agent, which slows down the pace of reaction overall^36^.

##### The impact of initial COD concentration

In this study, the impact of starting COD concentrations on the reduction of organic materials represented by COD from synthetic wastewater was examined (200, 400, 600, 800, and 1000 mg/L), as shown in Fig. 4d. The COD removal rate percentages were (90, 89.5, 84.2, 82.8, and 79%). The pH was adjusted using solutions of 0.1 M sulfuric acid and 0.1 M NaOH. 0.1 g of FeSO_4_.7H_2_O was added to each beaker. It employed the Jar test. The three minutes of mixing at 150 rpm, the 30 min of settling, and the subsequent filtering were all completed at the time of contact. The relationship between initial concentrations and elimination percentages was used to determine the optimum concentration for the chosen dose^36^.

#### Reduction of organic materials represented in COD by using coagulation process (step b)

##### Coagulant selection

**Figure 5:**
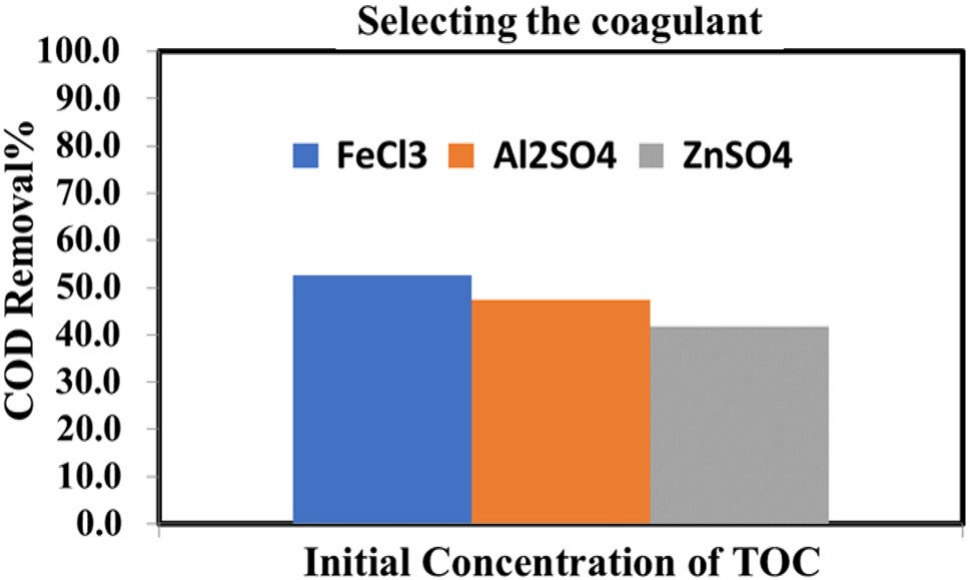
Impact of different coagulants on COD Removal%.

In coagulation techniques, the removal of organic materials represented by COD from synthetic wastewater was studied by using different common coagulants. Various types of coagulants were used, namely Al_2_ (SO_4_)_3_, FeCl_3_, and ZnSO_4_. According to the results, the COD percentage values were 52.6, 47.4, and 41.7%, respectively as illustrated in Fig. 5. The findings demonstrated that FeCl_3_ eliminates significant quantities of pollutants when compared to other coagulants.

##### The impact of pH on COD reduction

**Figure 6:**
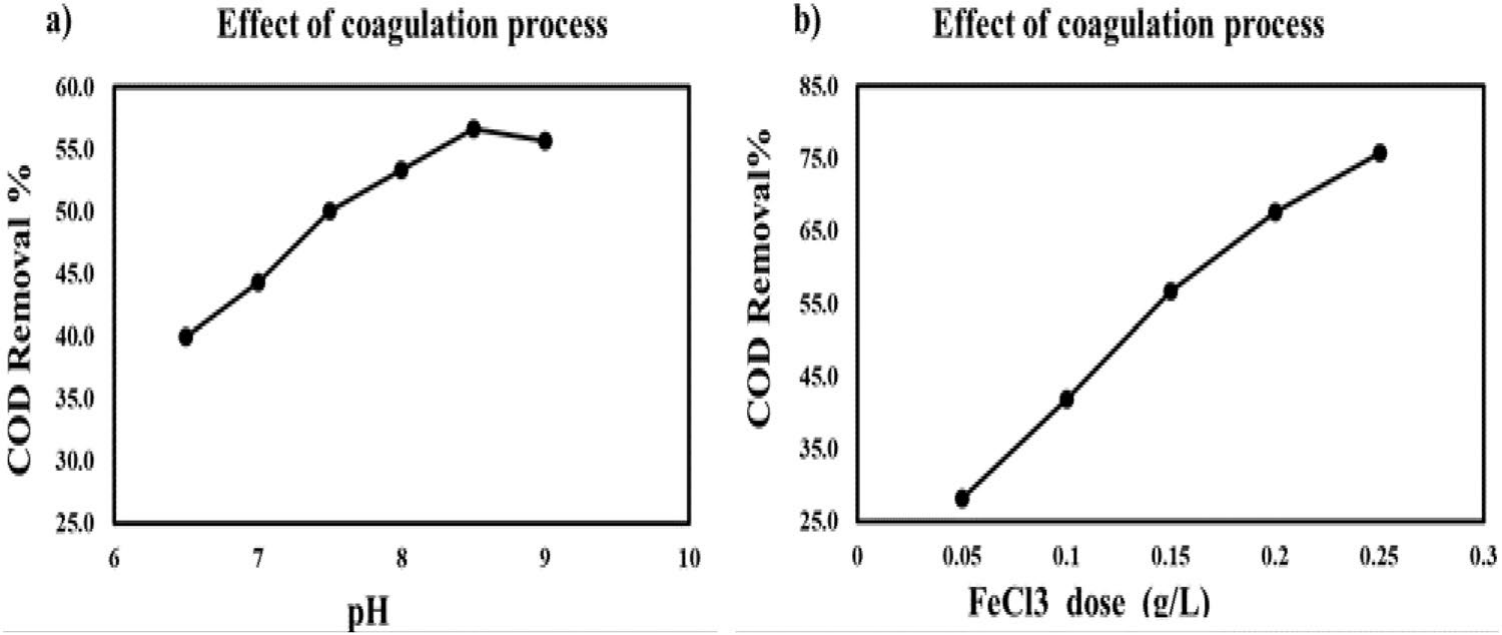
Impact of operating parameter on COD removal using coagulation process (**a**) pH, and (**b**) FeCl_3_ dose.

The most important factor in the coagulation process is pH Water's properties, the type of coagulant employed, and all other factors affect the pH^37^. The impact of pH was tested in the range of 6.5 to 9. FeCl_3_ is examined in six experiments at different pH values of 6.5, 7, 7.5, 8, 8.5, and 9, as illustrated in Fig. 6a. The strongest coagulant (FeCl_3_) was added with a sample of wastewater. The pH was adjusted using solutions of 0.1 M NaOH and 0.1 M sulfuric acid. The removal efficiency was 56.7% at the optimum pH of 8.5. The removal efficiencies are higher under alkaline conditions than under acidic conditions. In order to get rid of the organic contaminants, iron hydroxides create molecules on the solid surface that have hydroxyl groups^38^. In the pH range of 6.5–9.0, the concentration of contaminants lowered as the pH rose. When the pH is elevated above 8.5, the efficiency of the wastewater remains fixed. In the pH region is 6.5–8.5, FeCl_3_⋅6H_2_O and hydrogen ions interact to remove the organic materials COD, resulting in pollution removal, and above this, it is possible to produce negatively charged organic contaminants and electrostatically prevent adsorption. In this case, greater quantities of the metal cation (coagulant) will be required. The residual doses of the excess chemicals applied have an impact on the survive as well as the growth of fish in surface water, which may be hazardous to human health^39^.

##### Impact of coagulant dose

In the coagulation process, 0.1 M NaOH and 0.1 M sulfuric acid solutions were used to change the pH^39^. At various dosages of the coagulant (0.05, 0.1, 0.15, 0.2, and 0.25). Its removal efficiency of COD was (28.1, 41.9, 56.7, 67.6, and 75.7%), respectively, as illustrated in Fig. 6b. The higher the optimal dose of ferric chloride, the higher the removal rate for COD. If the dose of coagulant exceeds the optimum dose, it poses a risk to human health^39^. The amount of remaining iron in the supernatant decreased because of the precipitate (FeOH)_3_. It may have absorbed the iron as follows in Eq. (7). The COD removal efficiency with different doses of coagulant is illustrated in Fig. 6b.

7 $${\text{FeCl}}_{{3}} + {\text{ 3H}}_{{2}} {\text{O }} \to {\text{ Fe }}\left( {{\text{OH}}} \right)_{{3}} \downarrow \, + {\text{ 3H}}^{ + } + {\text{ 3Cl}}^{ - }$$

#### Reduction of organic materials represented in COD by using bimetallic (Fe/Cu) nano-particles (step c)

##### Impact of pH value on COD reduction

**Figure 7:**
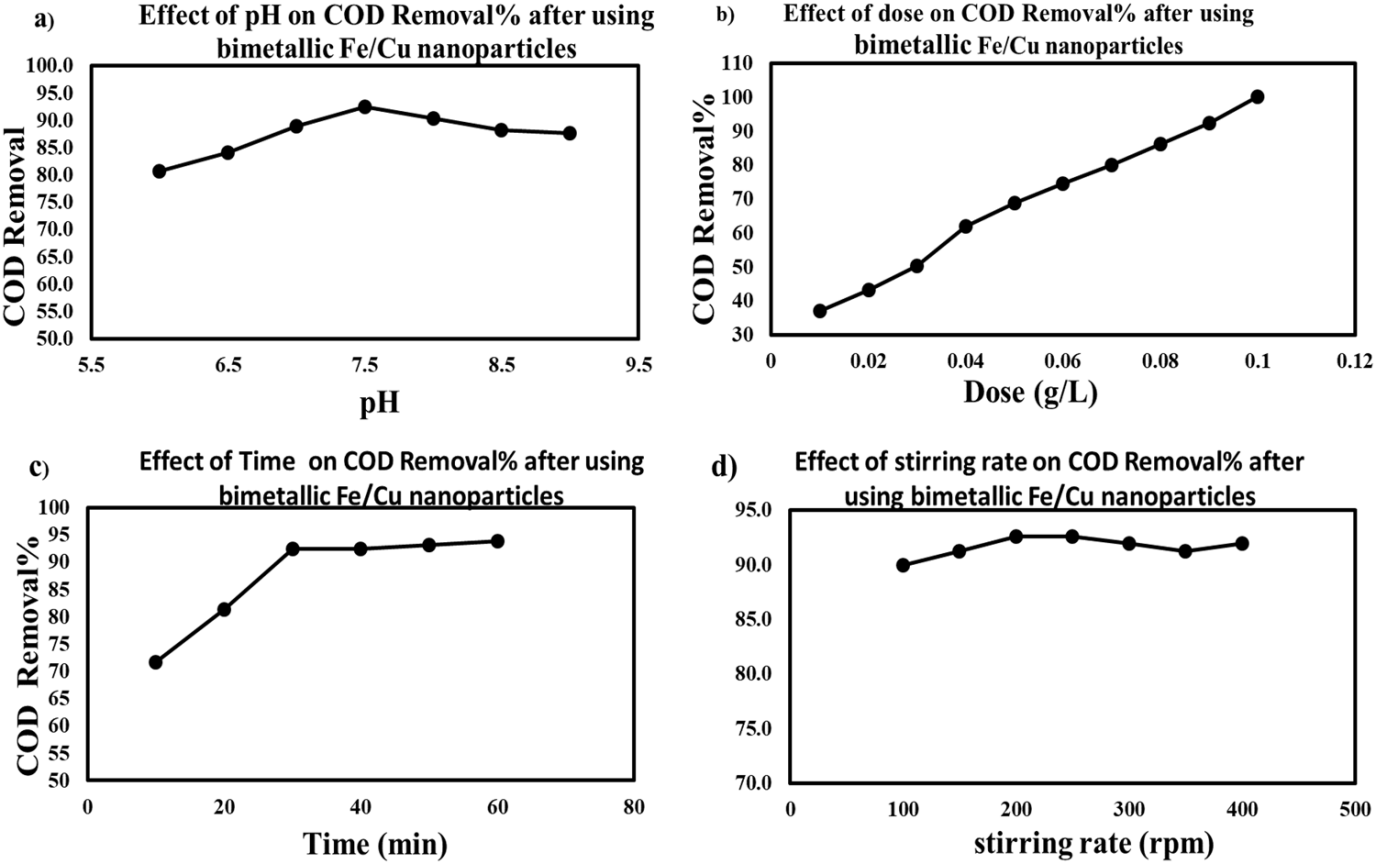
Impact of operating parameter on the removal of organic materials represented in chemical oxygen demand (COD) by using bimetallic Fe/Cu nanoparticles (**a**) pH, (**b**) dosage, (**c**) time, and (**e**) stirring rate.

According to the batch study, the impact of pH on acidic, neutral, and alkaline environments has been investigated for synthetic wastewater. The result showed that the greatest effectiveness of removal was 92.7% at pH 7.5, as illustrated in Fig. 7a. The greatest effectiveness of treatment was at pH levels between 7.5 and 8. Also, the lowest effectiveness of treatment was at high pH (acidic conditions) because the nanoparticles were dissolved because of acidic environments. This reduces the empty site of the sorbent nanoparticles, and thus the total adsorption activity of the sorbent nanoparticles decreases. In addition, nanoparticles' free electrons neutralize acid with H^+^, so their degradation properties decrease. In an alkaline medium, high OH^−^ and the free electrons of nanoparticles have an effective effect on adsorption ability, so the overall adsorption ability of nanoparticles is affected by the steric ions of the nZVI area and contamination causes. Because of their large surface area, nanoparticles are highly reactive.

##### Impact of adsorbent dose

In this study, the impact of bimetallic Fe/Cu nanoparticle dosage on the reduction of organic materials represented in COD from synthetic wastewater was studied (0.01 to 0.1 g/L). 0.1 M NaOH and 0.1 M sulfuric acid solutions were used to change the pH to 7.5 at 200 rpm at a concentration of 91 mg/L and a 30 min contact time, as illustrated in Fig. 7b. Increasing the adsorbent dosage increases the number of vacant adsorption sites, which increases the removal rate^40^. For the degradation process, when the adsorbent doses increase, the COD removal increases due to the increased vacant sites of sorbent nanoparticles and the free electrons of nanoparticles^41^.

##### Impact of time

According to the batch study, the impact of time on the reduction of organic materials represented in COD from synthetic wastewater was studied at (10, 20, 30, 40, 50, and 60) minutes and 0.09 g/L dosage of bimetallic Fe/Cu nanoparticle at pH 7.5 with a 30-min contact time, and the removal percentages were (71.6, 81.3, 92.4, 92.4, 93.1, and 93.8%), respectively, as shown in Fig. 7c. In this study, the COD removal percentage by using bimetallic nanoparticles (Fe/Cu) was 92.4% at 30 min. The impact of contaminants on the empty sites of the nanoparticles increased gradually with increasing time until an equilibrium condition was achieved^41,42^.

##### Impact of stirring rate

In this study, the impact of stirring rate on the reduction of organic materials defined by COD from synthetic wastewater was studied at 100, 150, 200, 250, 300, 350, and 400. By adding 0.09 g/L of bimetallic nanoparticle (Fe/Cu) at pH 7.5, the concentration was 91 mg/L at 30 min of contact time, and the removal percentage was 90, 91.3, 92.7, 92.7, 92, 91.3, and 92%, respectively, as shown in Fig. 7d. On bimetallic Fe/Cu nanoparticle surfaces, physical and chemical adsorption are both occurring; adsorption by chemical methods is more stable than adsorption by physical methods. In addition, the stirring rate can reduce the stability of chemical adsorption. Physical adsorption produces several molecules equal to the total amount of material adsorbed at each individual active site. The method of adsorption is based on the chemical reaction of contact between sorbents and adsorbents. As the adsorption process continued, the adsorption energy gradually decreased until it was eliminated. Consequently, the slight increase in elimination efficiency caused by stirring rate can be compared to the chemisorption reaction, and this correlates with the kinetic data^43^.

### Mechanism of degradation

Researchers describe how organic molecules are reduced by bimetallic nanoparticles. The activation energy of the organic compound reduction process is known to decrease when a second metal is present on the nZVI surface. Because of this increased interaction between the organic chemical and the nanoparticles, the velocity of the reaction is accelerated. The second metal often enhances the electron transit between nZVI and the target molecule. where nZVI acts as an electron donor and the second metal as an electron collector. Using nZVI, bimetallic nanoparticles efficiently break down organic molecules that usually break down more slowly. The degradation of organic compounds may be greatly impacted by the binding strength (metal–organic compound). The bimetallic nanoparticles are first coated with an organic chemical that causes the C–halogen link to break and hydrogen to replace the halogen. This is followed by the desorption of the freshly dehalogenated component. Bimetallic nanoparticle use might shorten this period^44^.

### Kinetic studies

**Figure 8:**
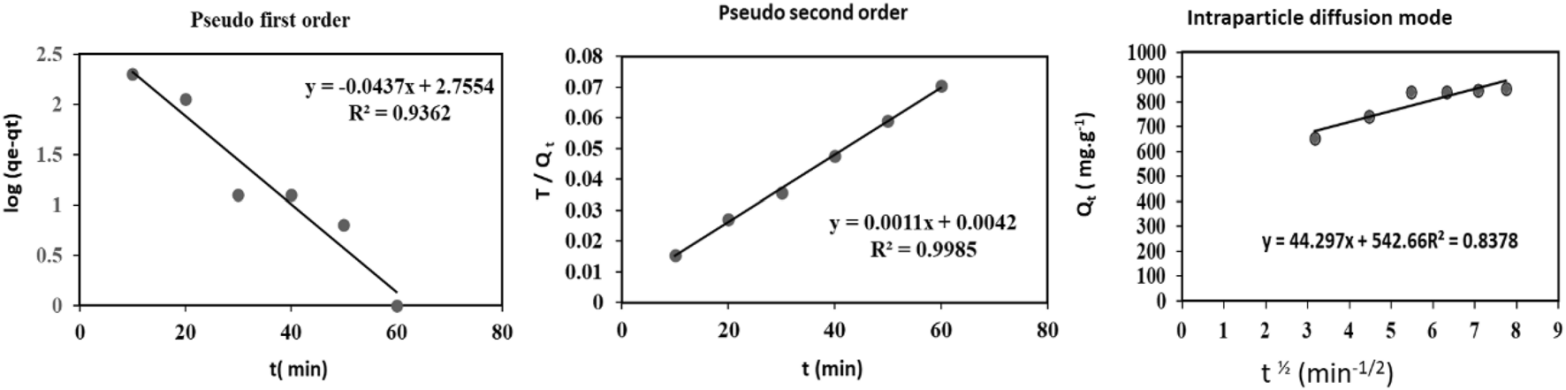
Effect of time and kinetic studies (pseudo first order, pseudo second order, and intraparticle diffusion mode).

The kinetic study is performed using pseudo-first-order model data (P.F.O.), pseudo-second-order model data (P.S.O.), and intraparticle kinetic model data to conduct kinetic research. The pseudo-second-order model data is more effective than the pseudo-first order and intraparticle diffusion models, with the highest correlation coefficients of R^2^. Kinetic studies for the adsorption of bimetallic Fe/Cu nanoparticles are shown in Tables 2 and Fig. 8^45^.

**Table 2:** Show the batch studies for the three stages of treatment.

No	Material selection	pH	Dose		Contact time (min*)*			Initial concentration (mg/L)
			a (g)	b (mL)	c	D	E	–
**Step A: fenton process**								
1	(FeCl_2_/H_2_O_2_)	1—6	0.05–0.30	2–12	3	10	30	200—1000
**Step B: coagulation—precipitation process**								
2	FeCl_3_	6.5–9	0.05–0.25	3	20	60	210	
3	Al_2_SO_4_							
4	ZnSO_4_							
**Step C: bimetallic Fe/Cu nanoparticles**								
5	Nano bimetallic Fe/Cu	6–9	0.01–0.1	–	–	–	30	91

a) Dose of Ferrous, b) Dose of H_2_O_2_, c) Mixing (min), d) Flocs formation (min), and E) settling time.

### Application to real tannery wastewater sample.

**Figure 9:**
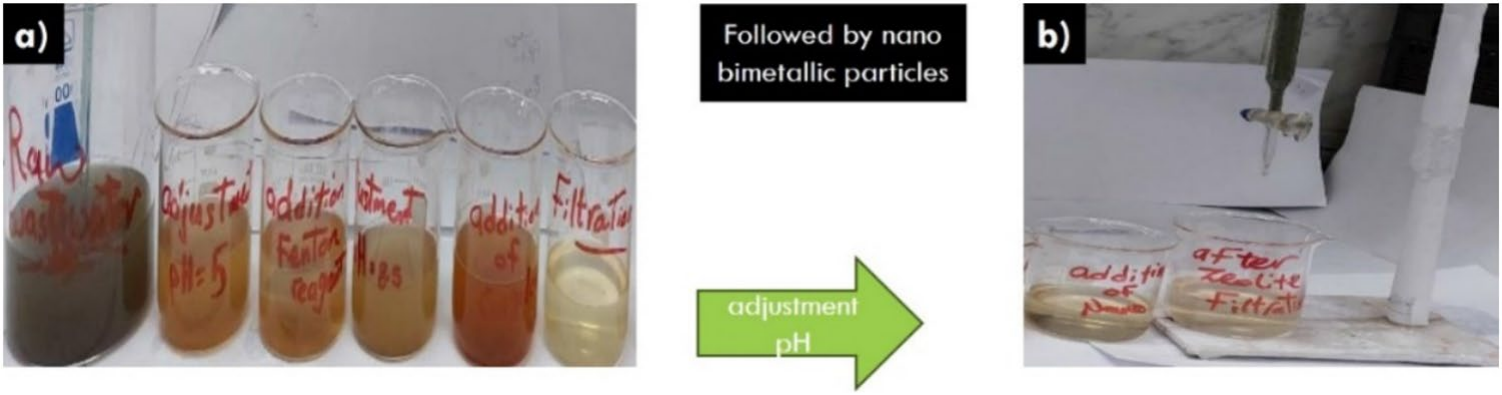
Shows stages of treatment of final discharge effluent by (**a**) using Fenton oxidation and coagulation- precipitation techniques and (**b**) using nano bimetallic particles.

**Figure 10:**
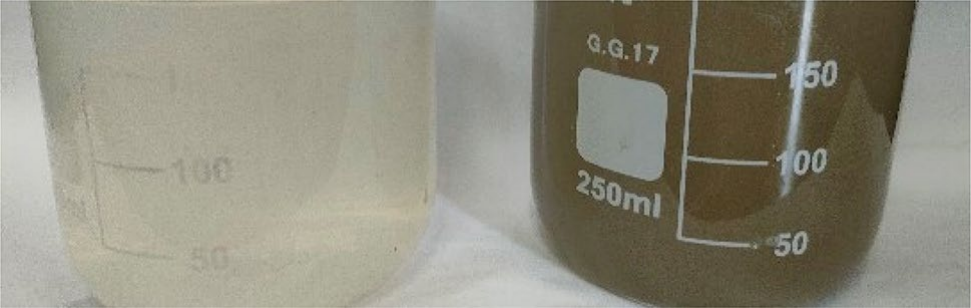
The final discharge effluent before and after stages treatment.

The above-mentioned treatment stages were applied to a real sample of the final effluent of raw industrial wastewater (tannery water) collected from the Bel Color Tannery in Quesna—Menoufia. It is considered one of the most dangerous pollutants that find their way into sewage when they are disposed of. The final effluent sample was analyzed before and after treatment. The results in Tables 3 and 4 showed the removal efficiency on the sample. All were efficient tests that complied with the Limits of executive Regulation of Law No.93 of 1962 as amended by Decree No.44 of 2000 concerning the discharge of final effluent to the public sewer system as illustrated in Figs. 9, 10^46^.

**Table 3:** Show kinetic studies for the adsorption of bimetallic Fe/Cu nanoparticles.

Kinetic model name	Parameters	Values
Pseudo-first-order K_1_	K_1_	0.1006411
	Qe (Cal)	3.1174
	R^2^	0.9362
Pseudo-second-order K_2_	K_2_	0.000327
	Qe (Cal)	909.09
	R^2^	0.9985
Intraparticle diffusion	K_id_	44.297
	C	542.66
	R^2^	0.8378

**Table 4:** Analysis of the sample from the final discharge effluent.

NO	Parameter	Results (ppm)	Results after treatment (ppm)	Removal %	Limits (ppm)
1	COD	5917	260	95	1100
2	BOD	3500	70	98	600
3	TSS	3600	77	97	800
4	TOC	1870	62	96	–
5	TKN	30.01	4.4	85	–
6	TP	3.1	0.3	90	25
7	CN-	0.018	0.006	66	0.2
8	Phenol	0.001˂	0.001˂	–	0.05
9	Chromium	0.4422	0.009	97	0.5
10	Oil & Grease	0.53	0.001	99	100

## Conclusions

In conclusion, the combination of advanced oxidation processes and nanotechnology has shown great potential for effective wastewater treatment. It offers opportunities to overcome the limitations of conventional treatment methods and achieve higher levels of pollutant removal. Nevertheless, further research and development are necessary to address the challenges associated with these technologies' scale-up, cost-effectiveness, and environmental impact. The marriage of advanced oxidation processes and nanotechnology has proven to be a winning combination in the realm of wastewater treatment. This study describes nanoparticles by using XRD, SEM, and EDAX analysis, which show the formation of nanoscale bimetallic Fe/Cu. Fenton oxidation reduces about 79% of the soluble COD standard, 1000 mg/L at acidic pH 5, using H_2_O_2_ and FeCl_2_ doses of 10 mL/L and 0.1 g/L, respectively. The maximum removal efficiency of COD, about 56.7%, was observed after using a coagulation dose of 0.15 g/L at pH 8.5. Finally, at using nano bimetallic Fe/Cu removed about 93% and was observed at pH 7.5 using a dose of 0.09 g/L, 30 min of contact time, and a stirring rate of 200 rpm. Overall, this study provides valuable insights into the potential of combining different treatment processes for wastewater treatment and could help inform research in this field in the future.

## Data Availability

All results of this paper are available if needed. If you want to request the data from this study, please contact with corresponding author.
